# Lumbar Scheuermann’s disease found in a patient with osteogenesis imperfecta (OI) caused by a heterozygous mutation in *COL1A2* (c.4048G > A): a case report

**DOI:** 10.1186/s12891-021-04401-7

**Published:** 2021-06-07

**Authors:** Shiwei Wang, Xiaoli Wang, Xiaochun Teng, Songbai Li, Hanyi Zhang, Zhongyan Shan, Yushu Li

**Affiliations:** 1 Department of Endocrinology and Metabolism, Institute of Endocrinology, NHC Key Laboratory of Diagnosis and Treatment of Thyroid Diseases, The First Affiliated Hospital of China Medical University, Shenyang, P. R. China; 2 Department of Radiology, The First Hospital of China Medical University, Shenyang, P. R. China

**Keywords:** Case report, Osteogenesis imperfecta, COL1A2 gene mutation, Scheuermann’s disease, Schmorl’s nodes

## Abstract

**Background:**

Osteogenesis imperfecta (OI) is a heterogeneous connective tissue disorder characterized by increased bone fragility and a series of extraskeletal manifestations. Approximately 90 % of OI cases are caused by type I collagen variants encoded by the collagen type I alpha 1 (*COL1A1*) or type I alpha 2 (*COL1A2*) gene. Lumbar Scheuermann’s disease is an atypical type of Scheuermann’s disease accompanied by Schmorl’s nodes and irregular endplates but without pronounced kyphosis. Although the etiology of Scheuermann’s disease is unclear, genetic and environmental factors are likely.

**Case presentation:**

Here, we report a 32-year-old male patient who experienced multiple brittle fractures. Gene sequencing revealed a heterozygous mutation, c.4048G > A (p.G1350S), in the *COL1A2* gene, and the patient was diagnosed with OI. Magnetic resonance imaging of his thoracolumbar spine revealed multiple Schmorl’s nodes.

**Conclusions:**

This is the first reported case of OI coexisting with the spinal presentation of Scheuermann’s disease. It is speculated that the *COL1A2* gene mutation might be an underlying novel genetic cause of Scheuermann’s disease. In conclusion, this case demonstrates the relationship between Scheuermann’s disease and OI for the first time and enriches the genotype-phenotype spectrum of OI.

## Background

Osteogenesis imperfecta (OI), also known as brittle bone disease, is a heritable skeletal dysplasia characterized by reduced bone mass, susceptibility to fracture, and variable associated connective tissue disorders. Extraskeletal manifestations include blue sclerae, hearing loss, dentinogenesis imperfecta, muscle weakness, scoliosis, joint laxity, easy bruising, and cardiac valve abnormalities [1]. Approximately 90 % of OI cases are caused by mutations in the type I alpha 1 (*COL1A1*) or type I alpha 2 (*COL1A2*) gene, which encode the α1 and α2 chains of type I collagen, respectively. Type I collagen is the most abundant protein present in the bone, skin, and tendon, and OI has become more fully understood as a predominantly collagen-related disorder [2].

Scheuermann’s disease, also known as Scheuermann kyphosis, is an osteochondrosis of the spine first described in 1921 [3]. Classic Scheuermann’s disease (type I) is characterized by painful, fixed, dorsal kyphosis occurring in adolescents, and radiographic manifestations include vertebral wedging, irregularity of the vertebral endplate, and Schmorl’s nodes [4]. In 1987, Blumenthal et al. first proposed lumbar Scheuermann’s disease (type II or atypical), which is characterized by Schmorl’s nodes and irregularity of the vertebral endplate but without severe clinical kyphosis [5]. The etiology of Scheuermann’s disease remains unclear, though it may be caused by excessive mechanical stress on a weakened vertebral endplate with defective growth. A hereditary component is considered to be a contributing factor [6].

Here, we report a patient with OI caused by a heterozygous mutation in the *COL1A2* gene (NM_000089): c.4048G > A (p.G1350S). Imaging of the thoracolumbar spine showed the characteristics of Scheuermann’s disease. To our knowledge, this is the first reported case of OI with Scheuermann’s disease presentation. The detected *COL1A2* gene mutation might be an underlying novel genetic cause of Scheuermann’s disease.

## Case Presentation

A 32-year-old man who experienced a recurrent fracture after sudden syncope was admitted to the First Hospital of China Medical University. Six months before the current time, he had sudden syncope while lying in bed; he felt low back pain after regaining consciousness. A computed tomography (CT) scan in the local hospital indicated multiple thoracolumbar fractures, and he was treated with external belt fixation. One month prior, he experienced another sudden syncope episode when he while sitting in the driver’s seat but before driving; he felt pain in his left shoulder upon waking. X-ray analysis indicated multiple fractures of the left proximal humerus, and he was treated with external fixation. He was then admitted to our hospital for further examination.

Laboratory assessment revealed elevated β-C-terminal telopeptides of type I collagen (β-CTX) (981.8 pg/ml; normal range<584 pg/ml) and N-terminal propeptides of type I collagen (P1NP) (81.93 ng/ml; normal range 20.00–80.00 ng/ml) and decreased 25(OH)D_3_ (12.99 ng/ml); serum calcium (2.3 mmol/L), phosphorus (1.27 mmol/L), magnesium (0.96 mmol/L), alkaline phosphatase (ALP) (93 U/L; normal range 35–100 U/L) and parathyroid hormone (PTH) (24.92 pg/ml; normal range 15.00–65.00 pg/ml) levels were normal. HLA-B27, arterial blood gas, kidney function, and liver function were all within normal ranges.

**Fig. 1:**
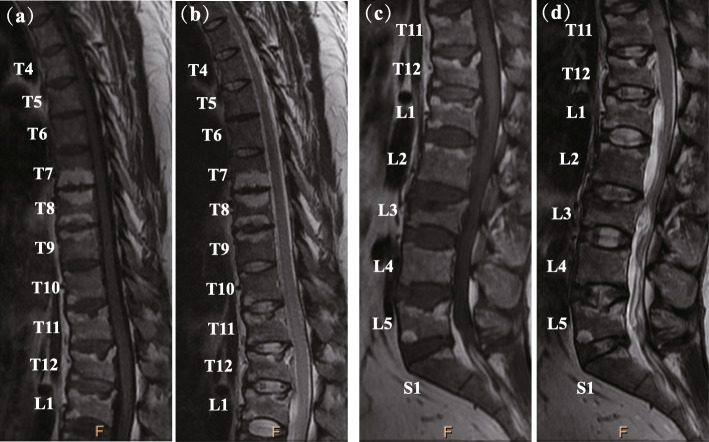
Magnetic resonance imaging (MRI) of the thoracolumbar spine. L3 vertebral compression fracture, local kyphosis, spinal canal stenosis, and cauda equina compression were observed. Vertebral endplate alterations were found in L1 to S1, and L2-S1 disc bulges and slight L4-S1 disc protrusions were noted. Schmorl’s nodes were observed in multiple thoracic and lumbar vertebrae (T8, T12, L1, and L5). (**a**) T1WI of the thoracic vertebra. (**b**) T2WI of the thoracic vertebra. (**c**) T1WI of the lumbar vertebra. (**d**) T2WI of the thoracic vertebra. T1WI, T1-weighted images; T2WI, T2-weighted images

X-ray radiographs of the skull, bilateral hands, left shoulder joint, and bilateral hips were performed. The skull image revealed a suspicious light transmission shadow at the left frontal bone. A bone island was detected in the left thumb proximal phalanx. The bilateral hips showed mild degenerative changes, and a comminuted fracture of the left greater tuberosity of the humerus were observed on X-ray and CT scans of the left shoulder joint. Magnetic resonance imaging (MRI) of thoracic and lumbar vertebrae is shown in Fig. 1, revealing L3 vertebral compression fracture, local kyphosis, spinal canal stenosis, and cauda equina compression. Vertebral endplate alterations were found in L1 to S1, and L2-S1 disc bulges and slight L4-S1 disc protrusions were noted. Schmorl’s nodes were observed in multiple thoracic and lumbar vertebrae (T8, T12, L1, and L5). Although bone mineral density (BMD) examined by dual-energy X-ray absorptiometry (DXA) of the hip joint was normal, the L2 (0.820 g/cm^2^, Z-score − 1.4) and L4 (0.877 g/cm^2^, Z-score − 1.7) BMD values were low.

The electrocardiogram performed indicated sinus tachycardia, and his heart rate was 107 beats/min. Cardiac ultrasound showed ventricular septum hypertrophy (9–11 mm), but left ventricular systolic function was normal in the resting state. CT angiography of the head and neck arteries indicated thinning of the right vertebral artery.

The patient had a 4-year history of hypertension. The patient had not suffered from any fracture in the past. His father had hypertension and cerebral thrombosis, and he had a 9-year-old son with Kawasaki disease. His mother and sister were healthy. The patient presented with normal stature and appearance (height, 173 cm; weight, 72 kg; BMI, 24 kg/m^2^; lack of blue sclera). The patient also had normal teeth and hearing, and no obvious kyphosis was observed.

### Genetic testing and mutation analysis

**Fig. 2:**
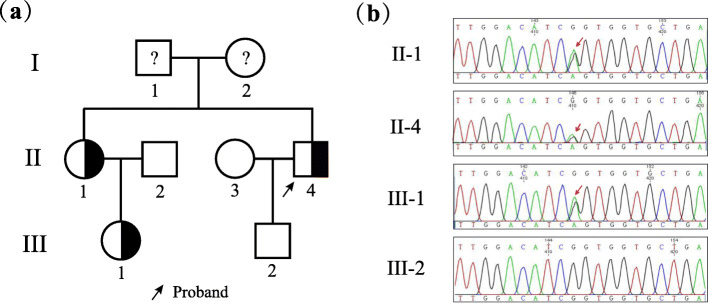
Pedigree and genetic analysis of the *COL1A2* gene in the proband and his family. (**a**) Pedigree. (**b**) Mutation analysis revealed a heterozygous mutation of the *COL1A2* gene, c.4048G > A (p.G1350S), in the proband (II-4). The proband’s sister (II-1) and her daughter (III-1) harbor the same mutation. The proband’s sister was an asymptomatic heterozygous carrier. The sister’s daughter had mild dentinogenesis imperfecta

Blood samples were collected from the patient and his family members, and genomic DNA was extracted using a blood extraction kit (Tian Jing Biochemical Technology Beijing, Ltd.). Gene regions, including splicing and coding regions of exons, of a panel of genes associated with bone metabolism were amplified by PCR and then sequenced using the Illumina NextSeq 500 system (Illumina, San Diego, CA, USA). Variations were confirmed by Sanger sequencing (ABI 3730, Applied Biosystems, Foster City, CA, USA). This analysis revealed a heterozygous mutation in *COL1A2* (NM_000089; c.4048G > A, (p.G1350S)) in the proband. Family investigation revealed that the proband’s sister and her daughter (his niece) carry this mutation. The proband’s sister was found to be an asymptomatic heterozygous carrier, and the niece previously experienced a fracture of the ankle and had mild dentinogenesis imperfecta without blue sclera (Fig. 2). Predictions for the c.4048G > A mutation in *COL1A2* are as follows: PolyPhen2 (Probably damaging, score = 1), FATHMM (Tolerated, score=-1.11), SIFT (Deleterious, score = 0.002) and PROVEAN (Deleterious, score=-4.84).

## Discussion and conclusion

Here, we describe a patient with OI caused by a heterozygous mutation in the *COL1A2* gene (NM_000089): c.4048G > A, (p.G1350S). Imaging of the spine showed the characteristics of Scheuermann’s disease.

OI, which is commonly known as brittle bone disease, is a relatively rare connective tissue disorder with significant bone manifestations. The prevalence of OI is one in 15–20,000 births [2]. Approximately 90 % of OI cases are caused by mutations in the *COL1A1* or *COL1A2* gene, leading to a decreased amount of normal type I collagen or production of type I collagen with abnormal structure. Type I collagen is the main protein component of the bone, skin, ligament, tendon, and many other connective tissues. Type I collagen is a heterotrimer containing two α1(I) chains and one α2(I) chain. It is synthesized as a procollagen molecule, and each proα(I) chain contains a typical triple-helical domain of more than 1,000 residues, consisting of a repeating Gly–Xaa–Yaa sequence, which is flanked by two globular extensions, the amino (N-) and carboxyl (C-) terminal propeptides. The two-terminal propeptides are cleaved after the triple helix is formed. Quantitative type I collagen defects often lead to mild OI, whereas structural defects can cause moderate to severe OI. The most common mutation causing structural defects is a single-nucleotide variant resulting in glycine replacement in the essential Gly–Xaa–Yaa triplets [1]. In the present case, mutation of the *COL1A2* gene occurred at nucleotide 4048, leading to glycine to serine alteration at p.1350, which is located in the C-terminal propeptide region (p.1120-p.1366). Fewer than 5 % of mutations occur in the C-propeptide domain of procollagen [7]. Once the C-propeptide trimer is folded, the triple-helical region folds in a zipper-like manner toward the N-terminus [8]. A previous study showed that the C-terminal propeptide of proα2(I) is crucial for efficient assembly of type I procollagen heterotrimers [7]. Compared with the proα1(I)-C-propeptide, pathogenic proα2(I)-C-propeptide variants are less common and generally associated with a milder form of OI. Cultured dermal fibroblast cells from patients with pathogenic proα2(I)-C-propeptide synthesize proα2(I) chains that are slow to assemble with proα1(I) chains to form heterotrimers and are retained intracellularly, which might be exposed to altered posttranslational modification. Some alterations, such as c.3952_3953 ins T and c.3487 T > C, lead to the uncharacteristic formation of proα1(I) homotrimers [9]. Compared with the heterotrimer, fiber formation by the homotrimer is impaired, with increased resistance to proteinases [8]. Overall, the mechanisms by which C-propeptide defects cause OI are not well understood. Possible explanations include a reduced type I collagen content in the extracellular matrix, delayed chain initiation and altered posttranslational modification of type I collagen, leading to decreased bone mineralization and bone strength; misfolded type I procollagen heterotrimers can be partly retained in cells, accumulating and increasing endoplasmic reticulum stress [7]. In the present case, the patient’s height was normal; despite generally normal bone imaging, he had multiple brittle fractures. Both heterozygous carriers, the patient’s sister did not exhibit any phenotype of OI, though her daughter experienced one fracture and had mild dentinogenesis imperfecta. Therefore, the *COL1A2* (c.4048G > A) mutation appears to be disease causing and result in mild type I OI in this pedigree.

Thus far, four cases of COL1A2 c.G4048A have been reported [10–12]. Yasuhiro Hamatani et al. described a 53-year-old male patient with the *COL1A2* c.G4048A mutation; diagnoses were OI and mucopolysaccharidosis type (MPS) III, though no gene mutation associated with MPS was discovered. The patient had a short stature, scoliosis, bone fractures, severe mitral regurgitation, and heart failure. Pathological analysis revealed acidic mucopolysaccharide accumulation and relatively few clumps of collagen fibers in the myocardial interstitium, valves, and aorta [10]. Thickening of the interventricular septum was also found in our patient. Nevertheless, considering a 4-year history of hypertension, it is unclear whether this change is related to the type I collagen variant. Huanzheng Li et al. used second-generation sequencing to carry out mutation detection and prenatal diagnosis in an OI family with *COL1A2* c.G4048A. The proband had a short stature, blue sclera, and fragility fractures. In contrast, our patient did not have a short stature or blue sclera, different from the above two cases. Another two cases (patient numbers #0001692 and #0002869) are present in Osteogenesis Imperfecta Variant Database (https://www.le.ac.uk/), though the related phenotypes are not reported. One patient (#0001692) was diagnosed with type I OI; however, changes were not found in the affected daughter. Therefore, the *COL1A2* c.G4048A mutation results in heterogeneous clinical manifestations in different families and different individuals in a given family.

As early as 1921, Scheuermann described a rigid kyphosis deformity of the thoracic or thoracolumbar spine, known as Scheuermann’s disease [3]. The prevalence of Scheuermann’s disease varies widely in different regions, ranging between 0.4 and 10 % [13]. Although the etiology of Scheuermann’s disease remains unknown, it shows a familial tendency, but the genetic pattern is not clear. Scheuermann’s disease may involve autosomal dominant inheritance. Some environmental factors, such as excess mechanical stress, might also play a role [4]. Lumber Scheuermann’s disease, also known as atypical Scheuermann’s disease or type II Scheuermann’s disease, was first described by Greene et al. in 1987 [5] and is characterized by significant Schmorl’s nodes and endplate irregularity at the thoracolumbar junction without severe clinical kyphosis [14]. A multicenter study reported that the prevalence of the lumbar type in Europe is 8 %, with no significant difference between the sexes [15]. Nevertheless, the prevalence in China has yet to be determined. Schmorl’s node is a herniation of the nucleus pulposus through the endplate into the adjacent vertebral body [16]. The primary function of the vertebral endplate, which is composed of a cartilaginous and an osseous component, is to prevent the intervertebral disc nucleus pulposus from being embedded in the vertebral body and at the same time has the effect of balancing and dispersing stress [17]. Histological studies of Scheuermann’s disease have revealed disorganized endochondral ossification, reduced collagen levels, and increased mucopolysaccharide levels in the vertebral endplate [4]. Some candidate pathogenic genes resulting in Scheuermann’s disease have been reported. For instance, a patient harboring an Arg^75^-Cys mutation in the collagen type II alpha 1 (*COL2A1*) gene was found to experience childhood-onset progressive osteoarthritis and vertebral changes, similar to Scheuermann’s disease [18]. Another mutation in the *COL2A1* gene (c.1636G > A, p.G546S) was detected in a girl presenting with hip dysplasia and Scheuermann’s osteochondritis [19]. The tryptophan allele of the collagen type IX alpha 3 (*COL9A3*) gene has also been associated with Scheuermann’s disease and intervertebral disk degeneration [20]. Bertrand Isidor et al. reported a boy with a de novo deletion of the Cullin 4B (*CUL4B*) gene, mutation of which commonly leads to syndromic X-linked mental retardation (XLMR). In addition to classic presentations of XLMR, such as mental retardation, minor facial anomalies, short stature, hypogonadism, and ataxia, the boy also displayed vertebral anomalies consistent with Scheuermann’s disease and aortic valvular dysplasia, which were new findings [21]. Although an association between Scheuermann’s disease and *COL1A1* and *COL1A2* was suspected, linkage analysis of three pedigrees failed to identify a relationship [22].

OI’ s spinal manifestations include scoliosis, kyphosis, craniocervical junction abnormalities, and lumbosacral pathology [23]. To our knowledge, presentations of Scheuermann’s disease have not been previously reported in OI. The *COL1A2* c.G4048A gene mutation may hamper the normal formation of type I collagen, affecting the ratio of mucopolysaccharide to collagen and rendering the defective endplates more prone to herniation. Therefore, it is speculated that a fraction of Scheuermann’s disease cases might have underlying dysplasia of bone or cartilage. As a form of skeletal dysplasia, OI might contribute to the occurrence and development of Scheuermann’s disease. Previous studies have indicated that Scheuermann’s disease has a strong association with inheritance, however, related pathogenic genes have not been fully uncovered. With the development of gene sequencing techniques, the pathogenic genes involved in Scheuermann’s disease warrant further study, which helps deepen our understanding of this disease.

We diagnosed this OI patient with Scheuermann’s disease presentation based on multidisciplinary cooperation, including endocrinology, orthopedics, and imaging. Although abnormal fragility fractures may suggest underlying skeletal dysplasia, they might not be given sufficient attention, especially if the fractures are not severe. This case report indicates that it is necessary to pay attention to a history of fragility fractures coexisting with abnormal bone imaging findings. If possible, a detailed examination should be carried out. Strengthening multidisciplinary cooperation may be helpful for the diagnosis and treatment of diseases.

There are some limitations to this article. First, we did not perform lumbar MRI for other family members. Second, experiments using cells to explore the function of the mutant protein were lacking. Therefore, we report this patient as a case report, and further investigation is necessary.

In conclusion, we report an OI patient with the heterozygous c.4048G > A (p.G1350S) mutation in the *COL1A2* gene who at the same time exhibited Scheuermann’s disease presentation. This is the first study to reveal the relationship between OI and Scheuermann’s disease. This case enriches the phenotype of OI and offers new insight into the genetic basis of Scheuermann’s disease.

## Data Availability

The authors declare that all data used during the study appear in the submitted article.

## Abbreviations

OI: Osteogenesis imperfecta
COL1A1: Collagen type I alpha 1
COL1A2: Collagen type I alpha 2
MRI: Magnetic resonance imaging
CT: Computed tomography
β-CTX: β-C-terminal telopeptides of type I collagen
P1NP: N-terminal propeptides of type I collagen
ALP: Alkaline phosphatase
PTH: Parathyroid hormone
BMD: Bone mineral density
DXA: Dual-energy X-ray absorptiometry
MPS: Mucopolysaccharidosis
COL2A1: Collagen type II alpha 1
COL9A3: Collagen type IX alpha 3
CUL4B: Cullin 4B
XLMR: X-linked mental retardation
